# Intestinal Subepithelial Myofibroblasts Support the Growth of Intestinal Epithelial Stem Cells

**DOI:** 10.1371/journal.pone.0084651

**Published:** 2014-01-06

**Authors:** Nan Ye Lei, Ziyad Jabaji, Jiafang Wang, Vaidehi S. Joshi, Garrett J. Brinkley, Hassan Khalil, Fengchao Wang, Artur Jaroszewicz, Matteo Pellegrini, Linheng Li, Michael Lewis, Matthias Stelzner, James C. Y. Dunn, Martín G. Martín

**Affiliations:** 1 Department of Bioengineering, University of California Los Angeles, Los Angeles, California, United States of America; 2 Department of Surgery, David Geffen School of Medicine at UCLA, University of California Los Angeles, Los Angeles, California, United States of America; 3 Department of Pediatrics, Division of Gastroenterology and Nutrition, Mattel Children’s Hospital and the David Geffen School of Medicine, University of California Los Angeles, Los Angeles, California, United States of America; 4 Stowers Institute for Medical Research, Kansas City, Missouri, United States of America; 5 Department of Molecular Cell and Developmental Biology, University of California Los Angeles, Los Angeles, California, United States of America; 6 Department of Pathology & Laboratory Medicine, University of Kansas Medical Center, Kansas City, Kansas, United States of America; 7 Department of Pathology, Veterans Affairs Greater Los Angeles Healthcare System, Los Angeles, California, United States of America; 8 Department of Surgery, Veterans Affairs Greater Los Angeles Healthcare System, Los Angeles, California, United States of America; Rush University Medical Center, United States of America

## Abstract

Intestinal epithelial stem cells (ISCs) are the focus of recent intense study. Current *in vitro* models rely on supplementation with the Wnt agonist R-spondin1 to support robust growth, ISC self-renewal, and differentiation. Intestinal subepithelial myofibroblasts (ISEMFs) are important supportive cells within the ISC niche. We hypothesized that co-culture with ISEMF enhances the growth of ISCs *in vitro* and allows for their successful *in vivo* implantation and engraftment. ISC-containing small intestinal crypts, FACS-sorted single ISCs, and ISEMFs were procured from C57BL/6 mice. Crypts and single ISCs were grown *in vitro* into enteroids, in the presence or absence of ISEMFs. ISEMFs enhanced the growth of intestinal epithelium *in vitro* in a proximity-dependent fashion, with co-cultures giving rise to larger enteroids than monocultures. Co-culture of ISCs with supportive ISEMFs relinquished the requirement of exogenous R-spondin1 to sustain long-term growth and differentiation of ISCs. Mono- and co-cultures were implanted subcutaneously in syngeneic mice. Co-culture with ISEMFs proved necessary for successful *in vivo* engraftment and proliferation of enteroids; implants without ISEMFs did not survive. ISEMF whole transcriptome sequencing and qPCR demonstrated high expression of specific R-spondins, well-described Wnt agonists that supports ISC growth. Specific non-supportive ISEMF populations had reduced expression of R-spondins. The addition of ISEMFs in intestinal epithelial culture therefore recapitulates a critical element of the intestinal stem cell niche and allows for its experimental interrogation and biodesign-driven manipulation.

## Introduction

The stem cell niche is a powerful model in our understanding of mammalian stem cell biology. Initially proposed to explain hematopoietic stem cell physiology [1], the niche model has been adopted to describe the various cellular and molecular players involved in intestinal epithelial homeostasis [2], [3]. Recent advancements include identification of putative intestinal epithelial stem cells (ISCs) and the development of culture methods for the long term propagation of intestinal epithelium *in vitro* [4]–[8].

Several candidate stem cell markers have been described recently [9]–[11], among which Lgr5 has received much attention. Through lineage tracing experiments, Lgr5 has been demonstrated to identify actively cycling crypt-base-columnar cells capable of elaborating all of the intestinal epithelial cell lineages [4]. Lgr5 is a co-receptor for the canonical Wnt pathway which is activated through the binding of R-spondin proteins [12]. Their binding results in LRP6 phosphorylation and increased beta catenin activity [13]. Wnt signaling has been shown to be essential for ISC maintenance, and its removal results in terminal differentiation and ultimate loss of the intestinal epithelium [14]. Recent advances in *in vitro* culture techniques have now made it possible to grow ISCs in the presence of exogenous growth factors, including the Lgr5 ligand R-spondin1 (Rspo1) [6], [15].

It has been suggested that some of these growth factors are derived from the neighboring Paneth cells *in vivo* [5], while others cues are likely provided by intestinal subepithelial myofibroblasts (ISEMFs) [16]–[18]. Paneth cells are tightly nested between ISCs in the crypt base; they act as anti-microbial defensive cells, while also producing supportive growth factors for neighboring ISCs [5]. It has been reported that Paneth cell-derived Wnt3 is necessary for *in vitro* intestinal epithelial cultures [19]. However, ISC niche maintenance *in vivo* is preserved even with conditional epithelial Wnt3 deletion [19] and Paneth cell ablation [20] – this suggests the presence of redundant sources of Wnt signaling from surrounding non-epithelial cells within the niche.

Among those cells in intimate contact with crypts *in vivo*, ISEMFs may serve as natural feeder cells for *in vitro* intestinal epithelial culture. Previous studies have shown that ISEMFs have a role in epithelial growth and differentiation [21] and facilitate *in vitro* growth of human enteroids [7]. However, the mechanism of their interaction with overlying epithelium remains poorly understood. In this study, we isolated murine ISEMFs and employed them in co-culture with ISCs to interrogate the nature of their interaction and identify responsible mechanisms. We hypothesized that ISEMFs provide necessary growth factors for the *in vitro* culture of intestinal epithelium derived from crypts and single ISCs and would further allow for their successful *in vivo* implantation.

## Materials and Methods

### Ethics Statement

Animal usage complied with regulations set by the University of California Los Angeles Chancellor’s Animal Research Committee and was approved as animal protocol number 2005–169. All efforts were made to minimize pain and suffering. Three mice strains were used for these experiments: C57BL/6-Tg(Actb-EGFP)1Osb/J (“eGFP”) (The Jackson Laboratory, Bar Harbor, ME), B6.129P2-*Lgr5^tm1(cre/ERT2)Cle^*/J (“Lgr5-eGFP”) (The Jackson Laboratory), and wild type C57BL/6 (Charles River, Wilmington, MA).

### ISEMF Isolation and Culture

ISEMFs were isolated from five-day old wild type C57BL/6 neonates using previously described methods [7], in which mesenchyme-rich small intestinal organoids are harvested using gentle enzymatic digestion and plated at a density of 5,000 per mL of ISEMF media. This media consisted of Dulbecco’s modified Eagle medium (DMEM)/Low Glucose/GlutaMAX (Invitrogen, Carlsbad, CA) with 10% FBS (Invitrogen), 1x Antibiotic-Antimycotic (Invitrogen), 0.25 U/mL insulin (Sigma, St. Louis, MO), 10 µg/mL transferrin (Sigma), and 20 ng/mL recombinant murine epidermal growth factor (EGF, Peprotech, Rocky Hill, NJ) [22]. After ISEMFs attached and formed colonies, they were subsequently passaged and expanded using standard cell culture techniques. In preparation for co-culture, ISEMFs were seeded into 48-well cell culture plates, with and without pre-treatment with gelatin, and allowed to grow to confluency. For conditioned media (CM) experiments, ISEMF media was replaced after the cells formed a confluent monolayer. The media was collected after 5 days of incubation, passed through a 0.2 µm pore size filter (Pall Corporation, Port Washington, NY), and used as ISEMF CM. The identity and purity of isolated ISEMFs was determined by quantitative real-time polymerase chain reaction (qPCR) and immunofluorescence (see below).

Neonatal murine ISEMFs were employed in the majority of experiments. Adult murine ISEMFs were identically isolated and grown from 2-month-old C57BL/6 wild-type mice in order to compare ISEMF populations.

### Intestinal Crypt Isolation and Culture

Small intestinal crypts were isolated from adult eGFP mice ranging 6–10 weeks old, using a previously described protocol [8]. Crypts were initially resuspended in 5 mL of Basic Crypt Media (BCM) consisting of Advanced DMEM/Ham’s F-12 (Invitrogen) with 1x Antibiotic-Antimycotic, 2 mM Glutamax (Invitrogen), and 10 mM HEPES (Invitrogen). Aliquots were microcentrifuged to yield crypts pellets for culture purposes.

Crypts were plated at a concentration of 250 crypts per 25 µL of Matrigel (BD Biosciences, Bedford, MA) into a 48-well plate, either directly into the well as a monoculture or on top of a confluent monolayer of ISEMFs. In order to investigate the effects of spatial separation, a subset of experiments were performed by placing 500 crypts in 50 µL of Matrigel on a trans-well cell culture membrane hanging over a 24-well companion plate (BD Biosciences), with or without a confluent layer of ISEMFs in the well below the membrane. The distance of separation between the trans-well membrane and the ISEMFs was 0.8 mm.

After allowing the crypt-Matrigel suspension to solidify for 10 minutes, cultures were treated with Complete Crypt Medium (CCM) consisting of BCM with 1x N2 (Invitrogen), 1x B27 (Invitrogen), 50 ng/mL EGF, 1 mM N-acetylcysteine (Sigma), 100 ng/mL recombinant murine Noggin (Peprotech), and 500 ng/mL recombinant human R-spondin1 (Rspo1, R&D Systems, Minneapolis, MN). Crypts grown with ISEMF CM were given media composing of 50% ISEMF CM, 50% Advanced DMEM/Ham’s F12, and the aforementioned growth factors at their respective concentrations. Fresh EGF, noggin, and Rspo1 supplements were added every 2 days, and the entire culture medium was replaced every 4 days. Cultures were assessed visually as described in the *Enteroid Measurements* section below. Subsets were fixed in formalin and assessed histologically or were lysed using DNA and RNA stabilization buffers and assessed with qPCR (see below).

### Single Cell Isolation and Fluorescence-activated Cell Sorting (FACS)

Single cells were obtained from Lgr5-eGFP adult mice for stem cell sorting following a previously described method [23]. The cells were prepared similarly to the point of antibody staining.

Single cells were stained for 7-AAD (Invitrogen) and APC-Annexin V (Invitrogen). They were then sorted with gates excluding doublets and 7-AAD and Annexin V positive cells and selecting for Lgr5-eGFP+ cells. Sorted single ISCs were collected into CCM with 10 µM Y-27632 (Sigma). The cells were pelleted and resuspended in Matrigel containing 750 ng/mL EGF, 1.5 µg/mL Noggin and 15 µM Jagged-1 (Anaspec, Fremont, CA) [11]. The suspension was plated into a 96-well with or without ISEMFs. The plate was incubated at 37°C for 15 minutes to allow the gel to solidify; CCM with 10 µM Y-27632 and 100 ng/mL Wnt3a (R&D Systems) was then delivered. Fresh EGF, noggin, and Rspo1 were added every 2 days, and the medium was changed every 4 days. These cultures were assessed for enteroid growth visually using ImageJ (NIH, Bethesda, MD).

### Subcutaneous Implantation

Cultures of either crypts or single ISCs, with and without ISEMFs, were removed from cell culture wells after 7 days *in vitro*. These detached cultures were then placed into non-woven polyglycolic acid (PGA) scaffolds (Synthecon, Houston, TX) and implanted subcutaneously into syngeneic adult recipient C57BL/6 mice as previously described [8]. Recipient mice were sacrificed 28 days following implantation, and the explants were formalin-fixed and processed histologically (see below).

### Histology

*In vitro* cultures and *in vivo* explants were fixed and processed for paraffin embedding as previously described [8]. Immunohistochemical staining was performed to identify α-smooth muscle actin (α-SMA), E-Cadherin, caudal type homeobox 2 (Cdx2), lysozyme, synaptophysin, and periodic acid-Schiff (PAS) positive cells (all reagents from Dako, Carpinteria, CA).

*In situ* immunofluorescence staining of the ISEMFs was performed after overnight fixation with 10% buffered formalin. The cells were permeabilized with 0.5% Triton-X (Sigma) in Tris buffer solution and washed with 0.05% Tween 20 in PBS (EMD Chemicals, Philadelphia, PA). Primary antibodies against α-SMA (Dako), desmin (Dako) and vimentin (Abcam, Cambridge, MA) were used at 1∶50 dilutions and incubated over night at 4°C. Corresponding AlexaFluor-488 conjugated secondary antibodies (Invitrogen) were used at 1∶200 dilutions and allowed to incubate for 30 minutes at room temperature. Nuclei were counterstained with Vectashield with DAPI (Vector Labs, Burlingame, CA). Images were acquired on a Nikon Eclipse Ti microscope (Nikon Instruments, Inc., Melville, NY).

### Enteroid Measurements

Micrographs of *in vitro* cultures were taken after 7 days using a Leica SP2 MP-FLIM microscope (Leica Microsystems, Wetzlar, Germany). The images were analyzed using the ImageJ image processing platform. The total number of viable enteroids per culture was counted, and their total and average cross-sectional area in the optical plane of focus was measured. Enteroid viability was defined by the presence of visually sharp borders along their basolateral (anti-luminal) side. Enteroid forming efficiency was computed as the proportion of initial crypts plated that yielded viable enteroids at 7 days.

### DNA/RNA Isolation and qPCR

DNA and RNA were extracted and purified from *in vitro* cultures on day 7 using the commercially available DNeasy and RNeasy kits (Qiagen, Valencia, CA), respectively. The qPCR reactions were prepared using the Quantitect Probe RT-PCR Kit (Qiagen). The primers and probe for eGFP-DNA were custom-designed and purchased from Eurofins MWG Operon, (Huntsville, AL) with 5′-ACTACAACAGCCACAACGTCTATATCA-3′, 5′-GGCGGATCTTGAAGTTCACC-3′, and 5′-(6-FAM)CCGACAAGCAGAAGAACGGCATCA(Tamra-Q)-3′ forward, reverse, and probe sequences, respectively. mRNA expression levels were assessed with reverse transcriptase qPCR, using commercially available primers and probes for target genes Acta2, Vim, Des, Lgr5, Cdx2, Lyz1, Chga, Muc2, and Vil1 (Applied Biosystems, Carlsbad, CA). The qPCR reactions were performed on an Applied Biosystems Prism 7900 Sequence Detection System. DNA qPCR results were normalized to the monocultures without ISEMF; RNA reverse transcriptase qPCR cycle numbers were analyzed according to the comparative C^T^ method using GAPDH as the housekeeping gene and whole small bowel as the tissue of reference [24].

### RNA Sequencing

Purified mRNA from supportive and non-supportive ISEMF populations were converted into cDNA libraries with the TruSeq RNA Sample Kit (Illumina, San Diego, CA), following the manufacturer’s protocol. The samples were run on the HiSeq 2000 sequencing system (Illumina) to obtain >65 million single end reads of 50 base pairs. The data was aligned to the mm9 assembly mouse genome (http://genome.ucsc.edu/cgi-bin/hgGateway?db=mm9) with the TopHat fast splice junction mapping software [25], using the Ensembl annotation (http://www.ensembl.org/) as an exon-exon splice junction library. Two mismatches and only unique hits were allowed for each read. HTSeq (EMBL, Heidelberg, Germany) was used to create a gene matrix by counting the number of reads for each gene and excluding any reads that did not uniquely map to a single gene. The length of each gene was calculated by taking the length of the union of all its possible exons. The RPKM (“reads per kilobase of exon per million reads mapped”) of each gene was calculated by dividing the number of reads mapped to it by both its length in kilobases and the library size in millions of reads. The gene matrix was analyzed with DESeq (Bioconductor, Seattle, WA) to estimate p-values for the comparison of the ISEMF samples. The scatter plot and heat maps were scaled by logarithmic transformation of the RPKM for all genes (scatter plot) and select genes of interest (heat maps).

### Data Analyses

Data were aggregated and expressed as mean values ± standard deviation. Statistical significance was determined with the Student’s t-test, and associated p-values were reported.

## Results

### ISEMF Characterization

**Figure 1:**
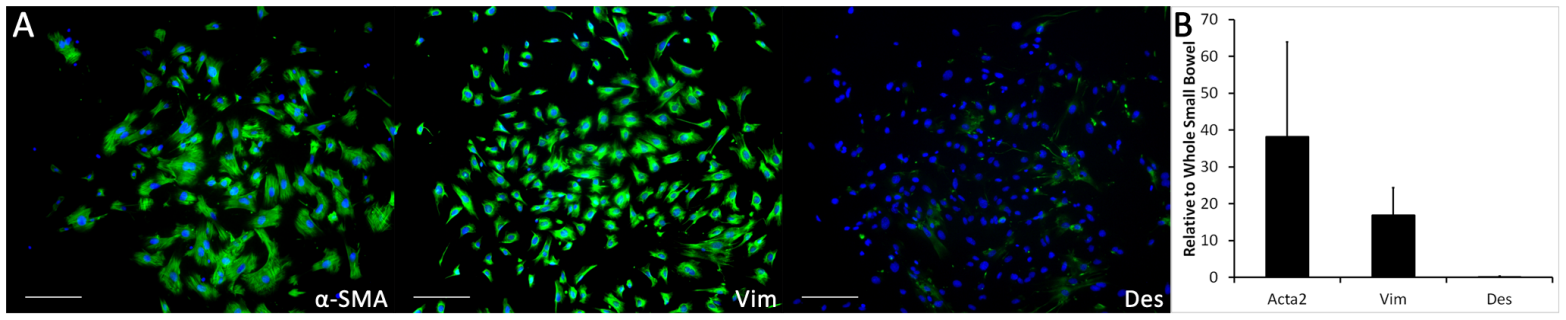
Characterization of intestinal subepithelial myofibroblasts (ISEMFs). Isolated ISEMFs were characterized through immunofluorescent staining and reverse transcriptase qPCR. (A) The cells were stained for α-smooth muscle actin (α-SMA, Acta2), vimentin (Vim), and desmin (Des). (B) Reverse transcriptase qPCR was performed on ISEMFs mRNA lysates using the same markers and normalized to small bowel (n = 4). The scale bar represents 200 µm.

The identity of isolated ISEMFs in *in vitro* culture was assessed through ISEMF-specific antibody staining and gene expression patterns. After isolation and expansion, ISEMFs appeared as elongated, spindle-shaped cells. These cells exhibited strong immunofluorescent staining against α-SMA and vimentin, but stained very weakly for desmin (Fig. 1A). Reverse transcriptase qPCR confirmed overexpression of Acta2 and vimentin relative to murine whole intestine, as well as negligible desmin expression (Fig. 1B). The expression pattern remained consistent for more than 10 passages. These data confirmed the anticipated phenotype of our isolated ISEMFs.

### Enteroid Growth from Crypts

**Figure 2:**
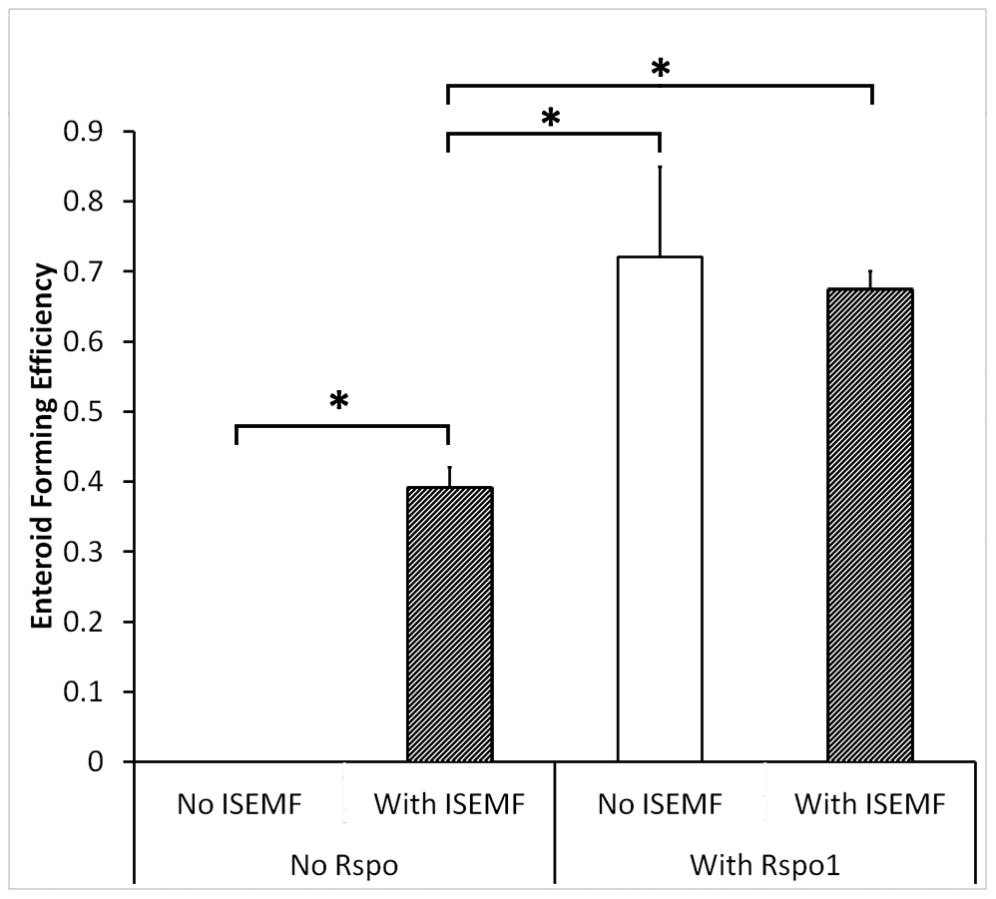
Enteroid forming efficiency from crypts after *in vitro* growth with and without ISEMFs. Enteroid forming efficiency was measured in mono- and co-cultures with ISEMFs, with and without exogenous Rspo1 in the media (n = 3). Asterisk indicates p<0.05 between the designated groups.

**Figure 3:**
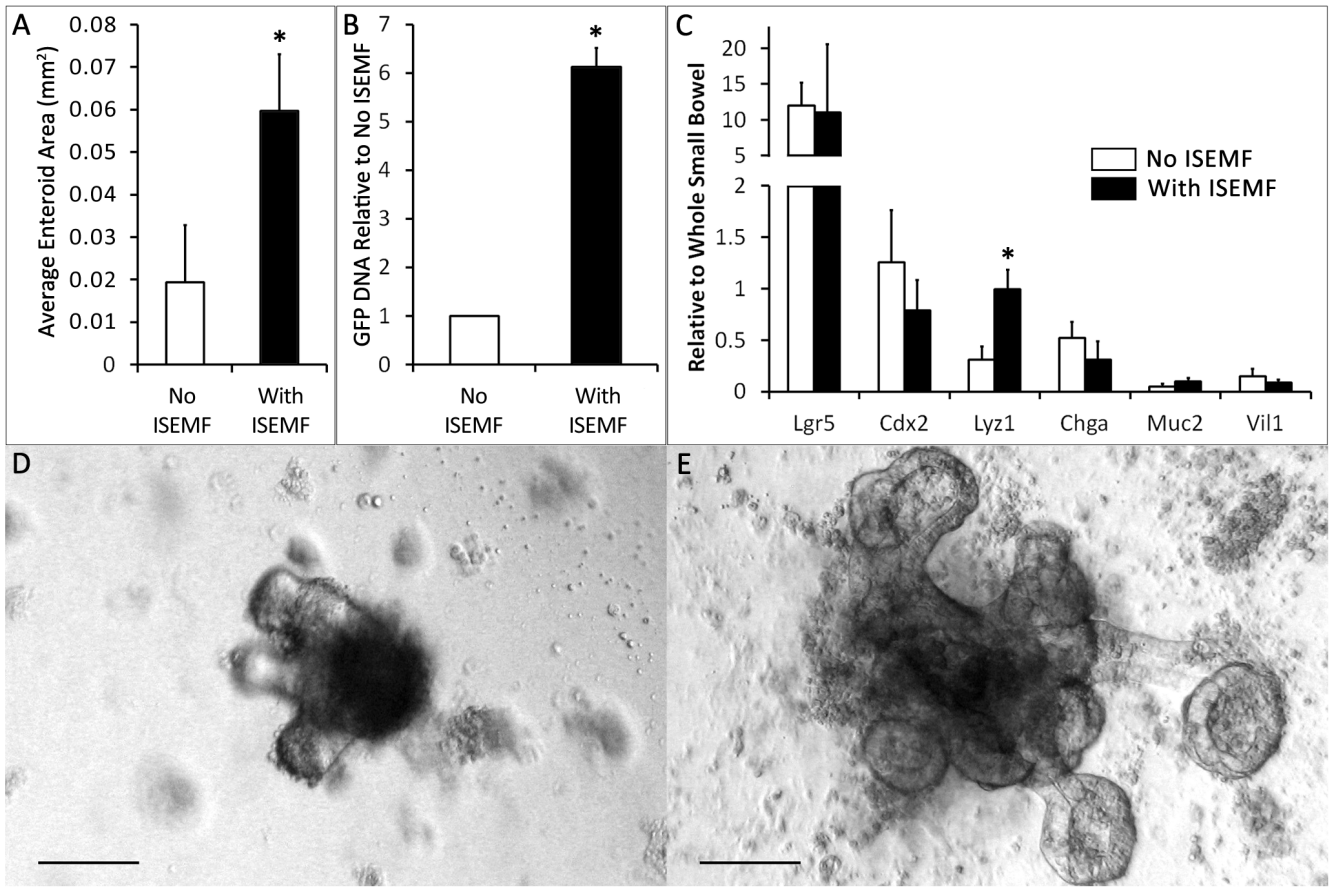
Epithelial characterization after *in vitro* culture with and without ISEMFs, in intimate contact. Crypts with and without ISEMFs were grown in culture media that contained Rspo1 and assessed after 7 days of culture. (A) Average area of an enteroid in co-culture was measured and compared with monoculture (n = 5). (B) eGFP DNA qPCR was used as a measurement of total epithelial expansion, normalized to the degree of expansion in monoculture (n = 7). (C) mRNA expression of Lgr5 and differentiated epithelial markers was assessed by qPCR and normalized to whole small bowel (n = 3). (D, E) Representative micrographs of an enteroid without ISEMFs (D) and with ISEMFs (E) were captured at day 7. The scale bar represents 100 µm. Asterisk indicates p<0.05 when compared to *No ISEMF*.

**Figure 4:**
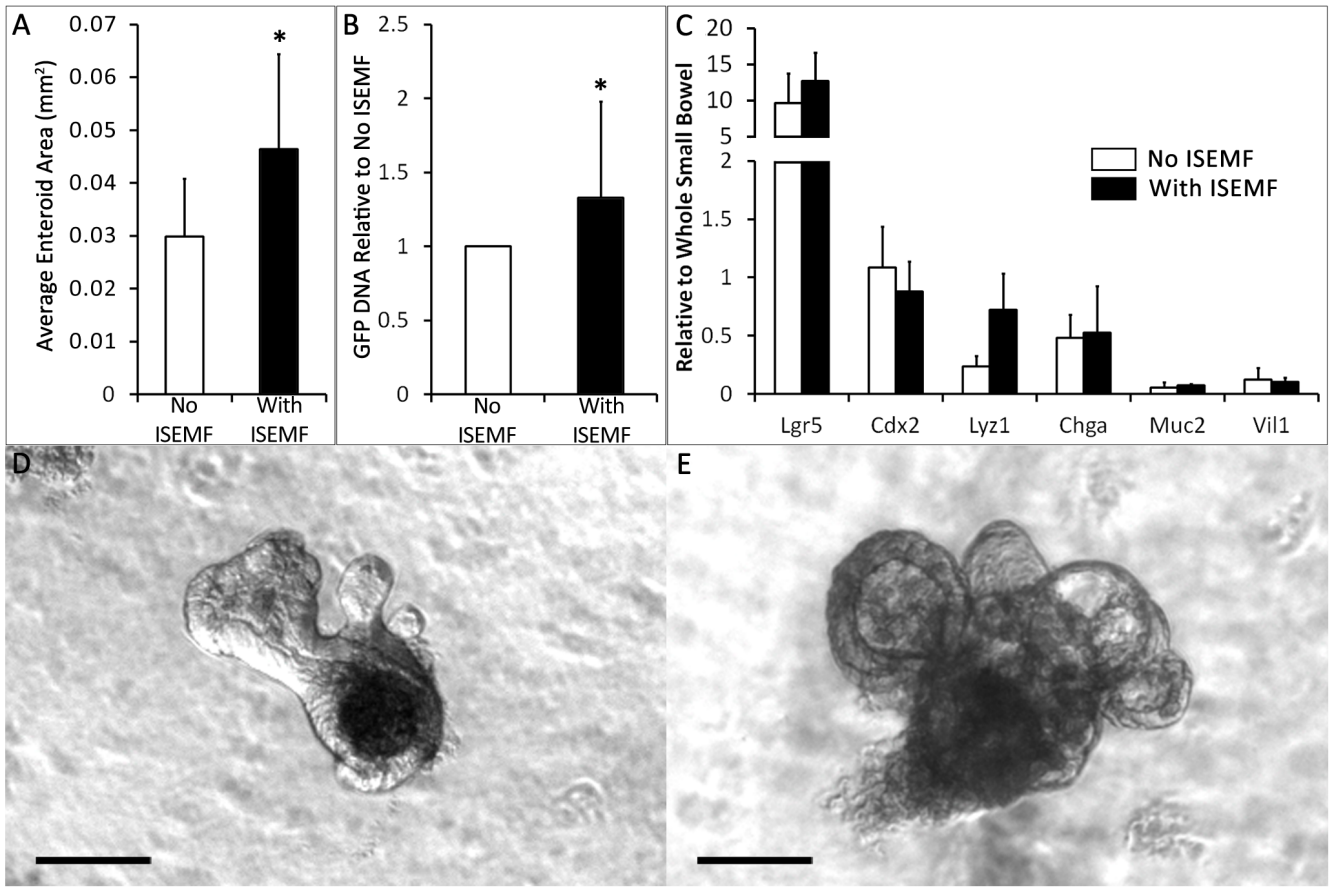
Epithelial characterization after *in vitro* culture with and without ISEMFs, separated by a trans-well membrane. Crypts with and without ISEMFs were grown in culture media that contained Rspo1 and assessed after 7 days of culture. (A) Average enteroid area was measured on cultures grown on a semi-permeable membrane, with or without ISEMFs in the well 0.8 mm beneath it (n = 5). (B) eGFP DNA was quantified with qPCR to measure epithelial growth (n = 7). (C) mRNA expression of Lgr5 and differentiated epithelial markers was assessed by qPCR and normalized to whole small bowel (n = 3). (D, E) Representative micrographs of an enteroid without ISEMFs (D) and with ISEMFs (E) below the membrane were captured at day 7. The scale bar represents 100 µm. Asterisk indicates p<0.05 when compared to *No ISEMF*.

To assess supportive effects of ISEMFs in intestinal epithelial culture, eGFP+ small intestinal crypts were cultured in the presence and absence of ISEMFs. When treated with media lacking exogenous Rspo1, crypt monocultures did not form enteroids and died within two days of plating, highlighting the previously described necessity for Rspo1 [15]. However, we found that crypts co-cultured with ISEMFs successfully formed enteroids even in the absence of exogenous Rspo1 (Fig. 2). After one week *in vitro* in the presence of Rspo1, co-culture in intimate contact with ISEMFs yielded enteroids approximately 3-fold larger (0.059±0.013 mm^2^ versus 0.019±0.013 mm^2^; p = 0.001), and containing 6.1±2.7-fold more eGFP DNA (p = 0.003) compared with monoculture with exogenous Rspo1 (Fig. 3A,B). Crypts formed budding enteroids in both conditions and had similar enteroid forming efficiencies (Fig. 2), but they were larger and more complex in the presence of ISEMFs (Fig. 3D,E). These beneficial effects were attenuated by physical separation: trans-well co-cultures that positioned both cell populations 0.8 mm apart rather than immediately adjacent to each other yielded enteroids only 1.5-fold larger than found in monoculture controls (0.046±0.018 mm^2^ versus 0.030±0.011 mm^2^; p = 0.03), with a similar decrease in the ratio of eGFP DNA expansion (Fig. 4A,B). These findings suggest that ISEMFs enhance the growth the ISC-containing crypts in a proximity-dependent fashion and supplant the necessity for exogenous Rspo1.

**Figure 5:**
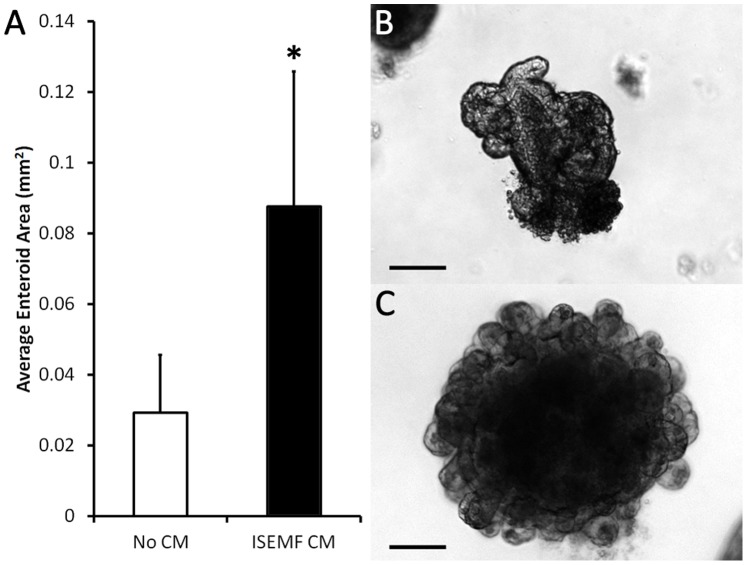
Epithelial growth after *in vitro* culture with and without ISEMF conditioned media (CM). Crypts were grown in culture media containing Rspo1, with or without ISEMF CM, and assessed after 7 days of culture. (A) Average area of an enteroid grown with ISEMF CM was compared to without conditioned media (n = 4). (B,C) Representative micrographs of an enteroid grown without CM (B) and with ISEMF CM (B). The scale bar represents 100 µm. Asterisk indicates p<0.05 when compared to *No CM*.

Crypts were grown with and without ISEMF CM to assess the necessity of interaction between ISEMFs and the epithelial cells. ISEMF CM was collected from confluent ISEMFs in monoculture after 5 days of incubation and filtered to eliminate cell contamination. Crypts grown with media containing ISEMF CM were 3 times larger (0.087±0.029 mm^2^ versus 0.038±0.016 mm^2^; p = 0.03) than those without conditioned media (Fig. 5A). These data suggest that the ISEMFs are actively secreting soluble factors that enhance ISC growth.

Messenger RNA from mono- and co-cultures was assessed through qPCR to determine the intestinal epithelial phenotype. The cultured enteroids expressed high levels of Lgr5 mRNA with both intimate contact and physical separation. There was not a significant difference in Lgr5 expression between enteroids cultured with and without ISEMFs (p = 0.9). The enteroids also expressed differentiated intestinal epithelial markers (Figs.3C, 4C). Lysozyme mRNA expression was 3-fold higher (p = 0.01) in intimately apposed co-cultures compared with monocultures (Fig. 3C); the remainder of the target genes demonstrated only non-significant differences between culture conditions. There were no significant differences in expression levels of any of the target genes between mono- and co-culture in the trans-well configuration (Fig. 4C).

**Figure 6:**
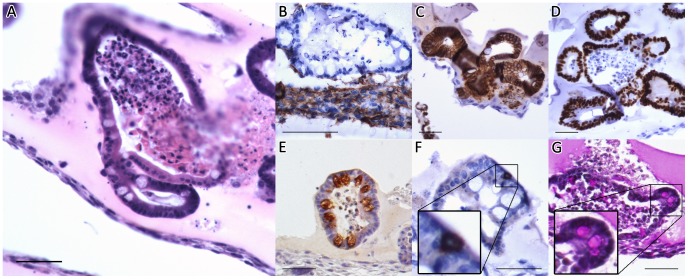
Histology of *in vitro* co-cultures on top of ISEMFs after 7 days. (A) Hematoxylin and eosin (H&E). (B–G) Immunohistochemical staining for (B) α-SMA, (C) E-Cadherin, (D) caudal type homeobox 2 (Cdx2), (E) lysozyme, (F) synaptophysin, and (G) periodic acid-Schiff (PAS). The scale bar represents 50 µm.

The presence of mature epithelium was also confirmed by immunohistochemistry (Fig. 6A,C–G). ISEMF (Fig. 6B) were identified surrounding enteroids comprised of E-cadherin (Fig. 6C), and Cdx2 (Fig. 6D) positive epithelium. Enteroids were further found to contain Paneth (Fig. 6E), enteroendocrine (Fig. 6F), and goblet cells (Fig. 6G). The qPCR and immunohistochemistry data suggest that while both mono- and co-cultures supported stem cells capable of giving rise to mature epithelium *in vitro*, lysozyme producing Paneth cells were more abundant when crypts were cultured in intimate contact with ISEMFs.

### In Vivo Implantation

Cultures were assessed for their long-term *in vivo* viability. Co-cultures on top of ISEMF spontaneously detached from the non-gelatinized wells. Crypts mono-cultured in Matrigel were mechanically detached. Both were placed on PGA scaffolds for subcutaneous implantation.

**Figure 7:**
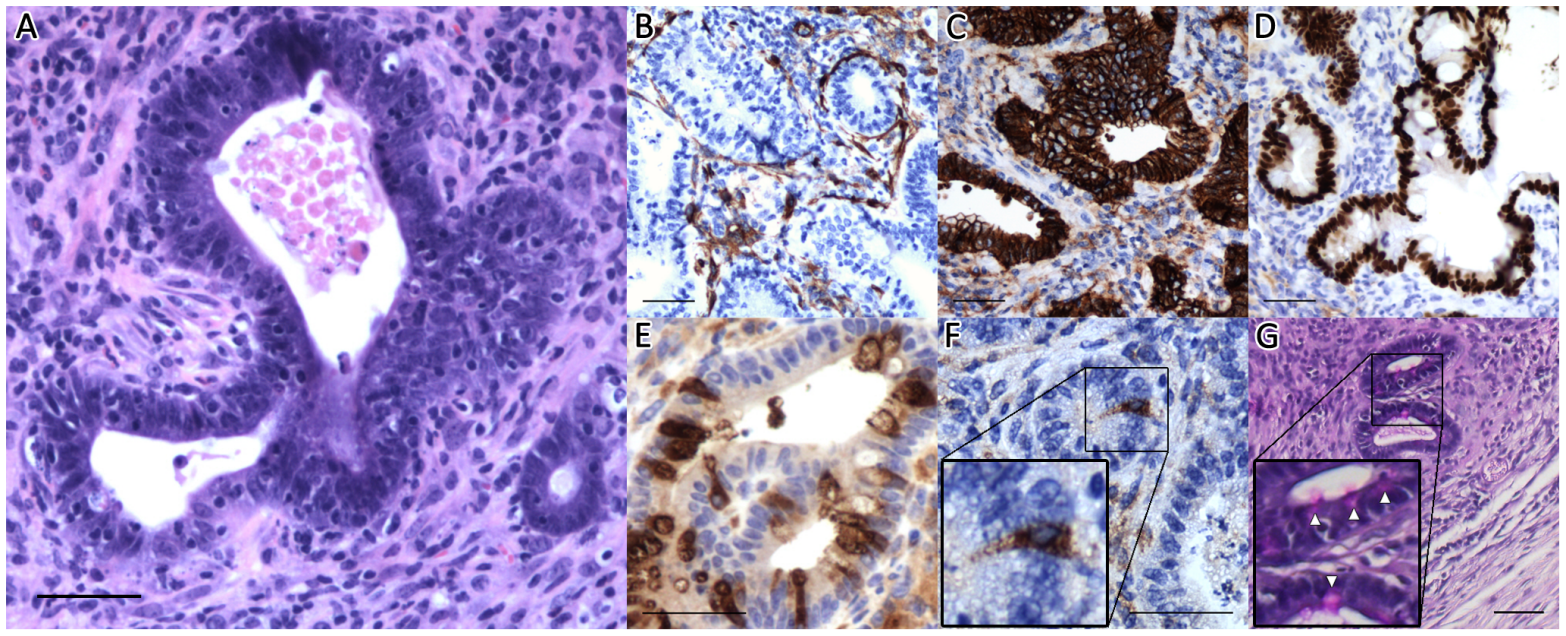
Histology of co-cultures implanted *in vivo* for 28 days. (A) H&E. (B) Immunohistochemical staining for (B) α-SMA, (C) E-Cadherin, (D) Cdx2, (E) lysozyme, (F) synaptophysin, and (G) PAS. The scale bar represents 50 µm.

After 28 days *in vivo*, histologic assessment of the retrieved co-cultures showed evidence of viable enteroids, with a preserved immunophenotype (Fig. 7A, C–G). Immunostaining further demonstrated that ISEMFs enveloped the engrafted enteroids (Fig. 7B). Implants containing only mono-cultured crypts without ISEMF consistently did not yield any epithelial cysts, thus confirming that ISEMFs are necessary to maintain the intestinal epithelium *in vivo*.

### Enteroid Growth from Single ISCs

**Figure 8:**
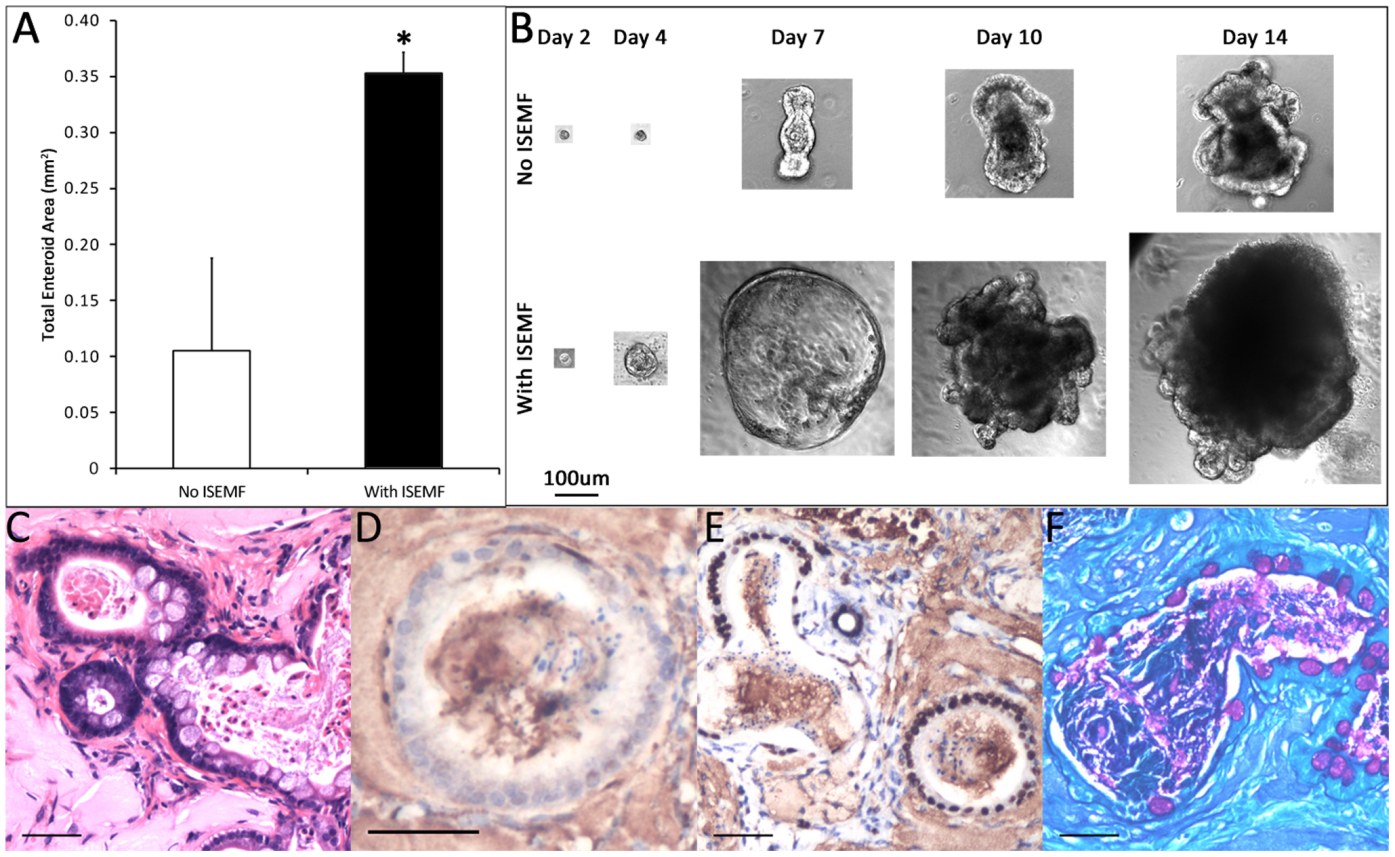
*In vitro* culture and *in vivo* histology of enteroids derived from single sorted stem cells. (A) The total cross-sectional area of the enteroids per well was measured to quantify the growth of the stem cells with and without ISEMFs (n = 2). (B) Time lapse images of the growth up to day 14. (C–F) Immunohistochemical staining of single ISC-derived enteroids and ISEMFs implanted *in vivo*. (C) H&E, (D) α-SMA, (E) Cdx2, and (F) PAS. The scale bar represents 100 µm in B and 50 µm in C through F. Asterisk indicates p<0.05 when compared to *No ISEMF*.

The beneficial effects of ISEMF co-culture was similarly assessed for intestinal epithelial cultures originating from single ISCs. Lgr5-positive single ISCs were isolated and FACS-sorted from an adult Lgr5-eGFP mouse, and they were grown in mono- and co-cultures as above. Resultant enteroids were larger and in co-culture compared with mono-culture. The total epithelial cross-sectional area per well was 3.5-fold larger with ISEMFs as compared to without (0.353±0.019 mm^2^ versus 0.105±0.083 mm^2^; p = 0.02) (Fig. 8A). Morphologically, the Lgr5-positive cells grew into budding enteroids in both conditions, but the ISEMFs again induced larger enteroids with more buds (Fig. 8B).

After 28 days of subcutaneous implantation, co-cultures of single ISC-derived enteroids and ISEMF were found in the retrieved explants (Fig. 8C), with ISEMFs surrounding the cysts (Fig. 8D). Cdx2 immunostaining confirmed the intestinal origin of the cysts (Fig. 8E) and PAS staining (Fig. 8F) suggested the presence of terminal epithelial lineage differentiation. The data suggest that ISEMFs improve the growth of single ISCs *in vitro* and maintain single ISC-derived intestinal epithelium *in vivo*.

### RNA Sequencing

**Figure 9:**
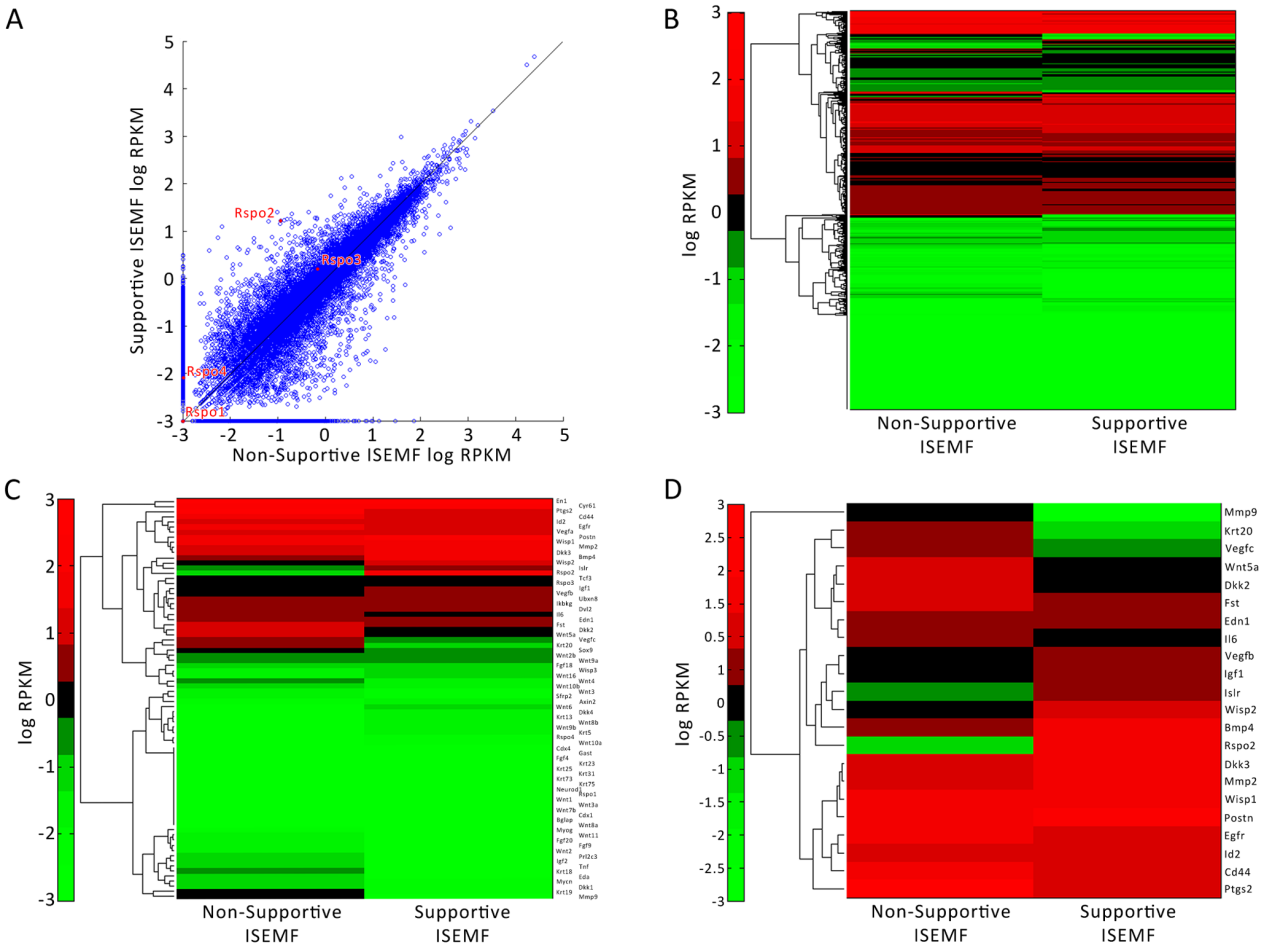
RNA whole transcriptome sequencing of supportive and non-supportive ISEMFs. The logarithmic transformation of RPKM values of (A) all genes, (B) genes coding for secreted proteins, and (C) genes coding for secreted proteins of the Wnt pathway were analyzed between supportive and non-supportive ISEMFs. (D) Select genes coding for secreted proteins of the Wnt pathway with at least a twofold difference in RPKM between supportive and non-supportive ISEMFs.

RNA sequenced whole transcriptomes of supportive and non-supportive ISEMFs were examined to identify potential mechanistic factors (GEO Accession GSE52402). Non-supportive ISEMFs were isolated from adult mice and demonstrated high levels of α-SMA and vimentin expression and low desmin expression (data not shown). They were found to only weakly support enteroid growth in the absence of exogenous Rspo1. A total of 22,379 protein-coding mRNA’s were read. Of these, 27.6% (6,179/22,379) were calculated to be differentially expressed between the supportive and non-supportive ISEMFs (Fig. 9A), using a standard p<0.01 criterion. 30.3% (1,197/3,949) of *secreted* protein-coding transcripts (Fig. 9B) and 41.0% (32/78) of secreted *Wnt pathway* protein-coding transcripts (Fig. 9C) were differentially expressed between supportive and non-supportive ISEMFs. Wnt pathway mediators such as the agonist Rspo2, antagonist Dkk3, and downstream product Wisp2 were found to have higher expression in the supportive ISEMFs (Fig. 9D). Rspo2 expression was markedly higher in the supportive ISEMFs compared to the non-supportive ISEMFs (RPKM 24.9 versus 0.0953; p = 10^−243^). Rspo- 1, 3, and 4 had a similar, low detection pattern between both ISEMF populations (Fig. 9A). The data suggest that while both ISEMFs exhibit similar overall gene expressions, the supportive ISEMF had higher expression in some Wnt pathway transcripts, for example Rspo2.

### Non-Supportive ISEMF

**Figure 10:**
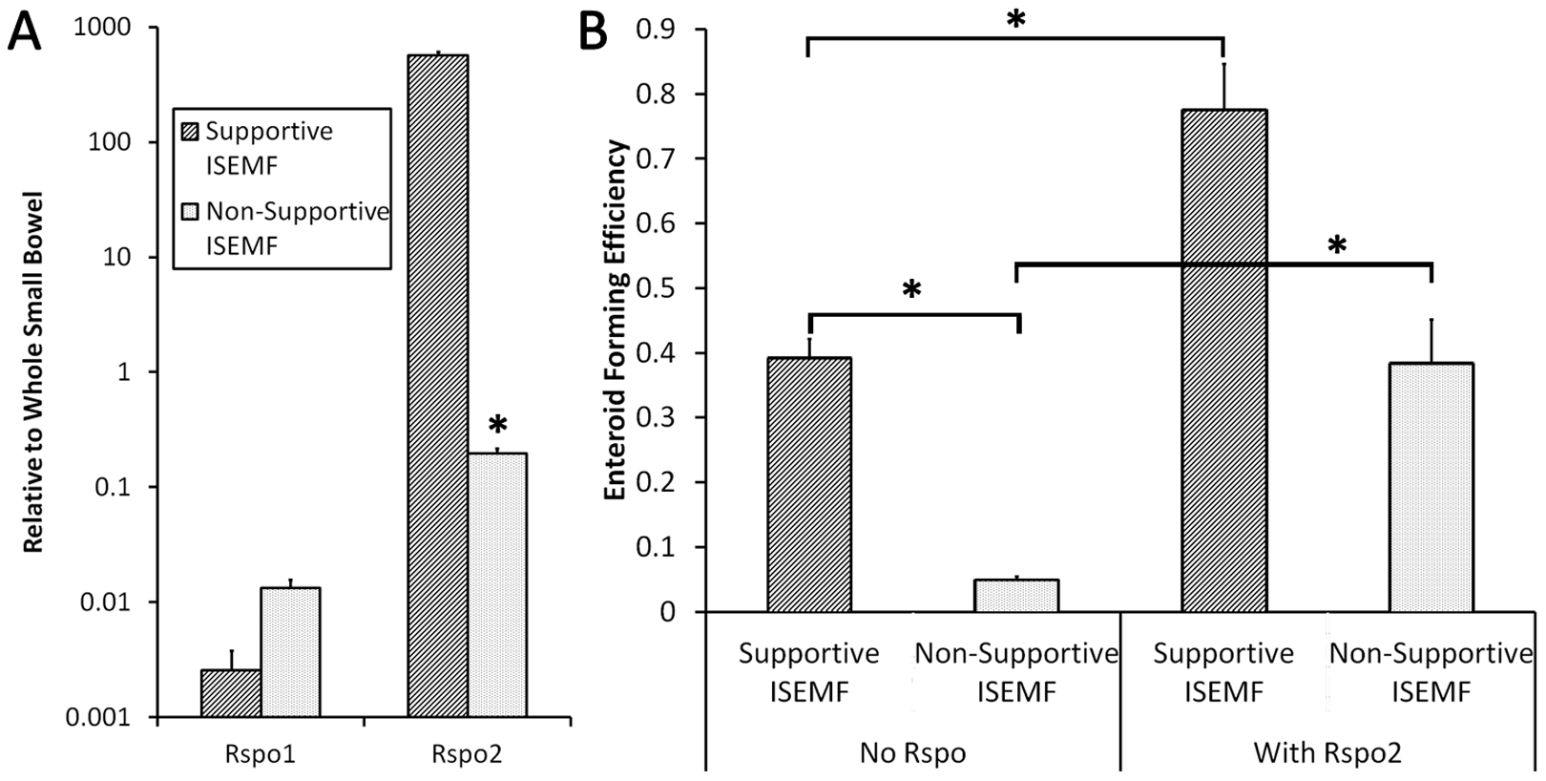
Supportive versus Non-Supportive ISEMFs. (A) Rspo1 and Rspo2 mRNA expression was measured by qPCR in ISEMFs that were supportive or non-supportive of crypt growth without exogenous Rspo1 (n = 3). (B) Enteroid forming efficiency was measured in co-cultures with supportive or non-supportive ISEMFs, with and without exogenous Rspo2 in the culture media. Asterisk indicates p<0.05 when compared to *Supportive ISEMF* in (A) and between the indicated groups in (B).

Supportive and non-supportive ISEMFs were examined in culture to further investigate the RNA sequencing finding of relative overexpression of Rspo2 in supportive populations. Subsequent qPCR confirmed that the supportive neonatal ISEMFs did not highly express Rspo1, but Rspo2 was highly overexpressed relative to small bowel. By contrast, non-supportive adult ISEMFs expressed low levels of Rspo2 (Fig. 10A). These cells were co-cultured in intimate contact with crypts, using culture media either devoid of R-spondin or containing Rspo2. In the stringent condition lacking exogenous R-spondin, we observed an improved enteroid forming efficiency with the supportive neonatal ISEMF compared with the non-supportive adult ISEMFs (39±3.0% versus 4.8±0.5%; p = 0.001) (Fig. 10B). When exogenous Rspo2 was added to the media, enteroid forming efficiency with the non-supportive ISEMFs improved to 38±6.8% (p = 0.01 compared with no Rspo2). The findings confirmed that Rspo2 is a critical mediator unique to supportive ISEMFs in our *in vitro* cultures.

## Discussion

In this study, we demonstrate that crypts or single intestinal epithelial stem cells co-cultured *in vitro* with intestinal subepithelial myofibroblasts give rise to larger enteroids than found in corresponding monoculture. The supportive ISEMF population was able to rescue crypts grown without exogenous Rspo1, a growth factor thought to be necessary for *in vitro* epithelial monoculture. Moreover, ISEMFs are capable of sustaining the growth of enteroids subcutaneously *in vivo* for one month without exogenous provision of growth factors. These findings suggest that ISEMFs play an important role within the ISC niche by providing growth factors beneficial to ISC maintenance and development.

It is likely that ISEMFs support the ISC niche through multiple mechanisms, including interaction through the Wnt pathway. The RNA sequences of the transcriptomes of supportive and non-supportive ISEMFs were highly concordant (Fig. 9A). However, key differences were observed in the genes coding for secreted Wnt proteins, such as R-spondin. Messenger RNA qPCR analysis of the ISEMFs showed negligible gene expression of Rspo1 compared to small bowel (Fig. 10A); however, Rspo2 was highly expressed in supportive ISEMFs. The latter has been reported to bind to Lgr5 and its homologues, which are components of the Wnt pathway [12]. Interestingly, Rspo2 has been found to be a more potent Wnt agonist than Rspo1 [26], which has traditionally been used in *in vitro* cultures [15]. *In vivo*, Rspo2 has been reported to enhance the growth of intestinal epithelial precursors and inhibiting differentiation [27]. Its regulation is vital for maintaining homeostasis in the intestinal epithelium.

Our prior *in vitro* data on human tissues revealed that ISEMFs from an infant were supportive of long-term epithelial growth, whereas ISEMFs from adults were non-supportive [7]. This observation motivated our efforts to isolate non-supportive ISEMFs from an adult mouse, in order to further elucidate the neonatal ISEMF support mechanism. The adult murine ISEMF population only weakly supported enteroid formation without added Rspo1; this was overcome by providing exogenous Rspo2. Intriguingly, whole jejunum from 1- or 6-month-old adult mice expressed 5 times less Rspo2 mRNA compared to week-old mice pups (Fig.S1). This finding begs the question of the role of ISEMFs in the adult ISC niche; it is unclear if there is an age-dependent shift in ISEMF phenotype. Further experiments are necessary to assess ISEMFs *in vivo* across a range of ages. R-spondin2 is a promising candidate responsible for signaling between ISEMFs and stem cells *in vivo*, and this possibility warrants further investigation.

Others have reported that ISEMFs provide growth promoters aside from R-spondins [16], [21]. Paneth cells have been suggested as important constituents of the ISC niche [5]. ISEMFs may further support ISCs by relatively favoring the Paneth cell lineage, as enteroids cultured with supportive ISEMFs expressed lysozyme more highly than those without ISEMFs (Fig. 3C). Therefore, ISEMFs both directly and indirectly provide the ISCs with sources of the Wnt signaling required for stem cell proliferation. These mechanisms remain poorly characterized and will require further elucidation in the future.

The capacity of ISEMFs to support growth in the absence of exogenous R-spondin is a significant advance in culture methodology. This appears to be a unique property of ISEMFs, as mouse embryonic fibroblasts did not support *in vitro* epithelial growth in the absence of Rspo1 (data not shown). Previously described culture methods require exogenous Rspo1 for crypt or stem cell survival [15], [23].

We chose subcutaneous rather than orthotopic implantation of enteroids in order to interrogate how faithfully our culture conditions reproduced the ISC niche. Successful *in vivo* intestinal epithelial engraftment was observed only with co-cultured ISEMFs and the recovered enteroids gave rise to differentiated epithelial lineages. We speculate that the inability to grow enteroids *in vivo* without ISEMFs is likely due to the absence of vital growth factors in the subcutaneous environment. Immunohistochemical staining for α-smooth muscle actin on the explants revealed that ISEMFs encircle the enteroids. The close proximity recapitulates the intestinal stem cell niche *in vivo*, where ISEMFs lie just basal to the crypts. Our *in vitro* findings with conditioned media suggest that ISEMFs are actively secreting growth-promoting soluble factors. However, direct comparison of distance in co-culture demonstrated an attenuated effect as the distance between epithelial and ISEMF compartments was increased, suggesting that close proximity between the ISEMFs and epithelial cells is preferable. The effect may be due to the concentration gradient of Rspo2 from the ISEMFs to the epithelial cells. However, it is still unclear if cell-cell contact is necessary *in vitro*. The close spatial relationship between ISCs and ISEMFs is likely a critical factor for *in vivo* survival in the absence of exogenous supplementation of growth factors.

Our findings support the use of ISEMFs in tissue-engineering applications. Previous studies from our laboratory have demonstrated that intestinal organoids containing crypts and surrounding mesenchyme are able to form a functional neomucosa *in vivo* [28]–[30]. However, the requirement for a substantial amount of harvested tissue to generate only modest quantities of neomucosa limits the clinical utility of this approach. In contrast, the present work demonstrates that it is possible to expand intestinal epithelial cell mass *in vitro*, while maintaining the capacity for successful *in vivo* engraftment. The addition of ISEMFs as a part of an experimentally recapitulated stem cell niche presents an exciting new avenue for intestinal tissue engineering.

### Conclusion

We conclude that co-culture of intestinal epithelial stem cells with ISEMFs yields larger enteroids with improved viability, even in the absence of exogenous growth factors. This effect allows for successful subcutaneous *in vivo* implantation and engraftment. ISEMFs appear to produce soluble factors that interact with the ISCs and promote their maintenance and self-renewal. Finding optimal conditions to integrate ISCs and ISEMFs into a functional tissue engineered neomucosa will be the next major challenge in developing translational clinical applications.

## Supporting Information

Figure S1 **R-spondin2 (Rspo2) mRNA expression in whole small intestine across several ages.** Whole (A) jejunum or (B) ileum from 7-day-, 1-month-, or 6-month-old mice were isolated and analyzed for Rspo2 mRNA expression through qPCR. The expression was normalized to the 7-day-old jejunum or ileum (n = 2). Asterisk indicates p<0.05 when compared to *7 Day Old Jejunum*.
(TIF)
