# Supplementary figures and images for: Intestinal Subepithelial Myofibroblasts Support the Growth of Intestinal Epithelial Stem Cells
Source: PLoS One. 2014 Jan 6;9(1):e84651. doi: 10.1371/journal.pone.0084651 (PMC3882257; doi:10.1371/journal.pone.0084651)

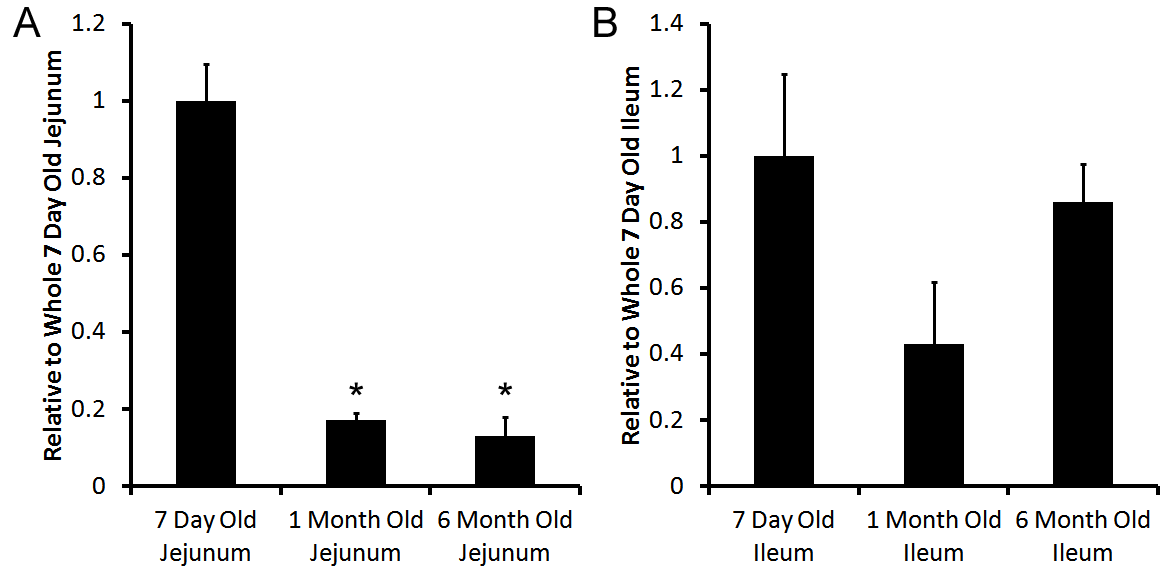

Supplement: Figure S1 — R-spondin2 (Rspo2) mRNA expression in whole small intestine across several ages. Whole (A) jejunum or (B) ileum from 7-day-, 1-month-, or 6-month-old mice were isolated and analyzed for Rspo2 mRNA expression through qPCR. The expression was normalized to the 7-day-old jejunum or ileum (n = 2). Asterisk indicates p<0.05 when compared to 7 Day Old Jejunum. (TIF) [file pone.0084651.s001.tif]
